# *Lobularia maritima* (L.) Desv. Aerial Parts Methanolic Extract: In Vitro Screening of Biological Activity

**DOI:** 10.3390/plants9010089

**Published:** 2020-01-10

**Authors:** Mariangela Marrelli, Maria Pia Argentieri, Pinarosa Avato, Filomena Conforti

**Affiliations:** 1 Department of Pharmacy, Health and Nutritional Sciences, University of Calabria, I-87036 Rende, Italy; mariangela.marrelli@unical.it; 2 Department of Pharmacy-Drug Sciences, Università degli Studi di Bari Aldo Moro, I-70125 Bari, Italy; mariapia.argentieri@uniba.it (M.P.A.); pinarosa.avato@uniba.it (P.A.)

**Keywords:** antioxidant, *Lobularia*, nitric oxide, obesity, pancreatic lipase, sweet alyssum

## Abstract

*Lobularia maritima* (L.) Desv. is a perennial herb growing wild in the Mediterranean basin. The aim of this work was to assess the fatty acid, terpene, phytosterol, and phenolic composition of the methanolic extract and its sub-fractions using Gas Chromatography-Mass Spectrometry (GC-MS), High-Performance Liquid Chromathography with Dioide-Array Detector (HPLC-DAD), High-Performance Liquid Chromathography-High Resolution Mass Spectrometry (HPLC-HRMS), and Electrospray Ionization Tandem Mass Spectrometry (ESI-MS/MS). The potential health benefits of this plant species have been investigated as well. The antioxidant activity was determined in vitro by means of 2,2-diphenyl-1-picrylhydrazyl (DPPH) and β-carotene bleaching tests. The inhibitory potential towards the production of the pro-inflammatory mediator nitric oxide was verified on lipopolysaccharide (LPS)-stimulated murine macrophage RAW 264.7 cell line. A remarkable inhibitory activity was observed for the dichloromethane fraction, with an IC_50_ value equal to 45.86 ± 1.05 μg/mL, a significant result if compared to indomethacin and the known nitric oxide synthase inhibitor N^G^-nitro-L-arginine methyl ester (L-NAME), used as positive controls. Moreover, the ethyl acetate fraction proved to be effective in inhibiting pancreatic lipase, an enzyme that plays a pivotal role in the gastrointestinal digestion of dietary fat, suggesting that this species could potentially be a promising source of useful compounds for the treatment of obesity.

## 1. Introduction

*Lobularia maritima* (L.) Desv. (Brassicaceae), commonly known as sweet alyssum, is a long-flowering plant, which produces abundant quantities of nectar and represents an excellent resource for several hymenopteran parasitoids [1,2]. This perennial herb occurs in coastal zones, dunes, and scrublands of the Mediterranean basin, but its flowering pattern is unusual for this area. The climate of the Mediterranean basin is characterized by a prominent seasonality, and most species reach their blooming peak in spring, with short flowering periods (usually 2 or 3 months). In contrast to this trend, *L. maritima* blooms for 10 months (from September to late June), with the peak of flowering in autumn [3].

This species is endemic to Italy [4], and the use of wild plants as a traditional food source in Southern Italy (Sicily) is documented [5]. 

Previous phytochemical investigation of *L. maritima* evidenced the presence of some interesting flavonoids, such as kaempferol, kaempferol-7-rhamnoside, kaempferol 3-glucoside-7-rhamnoside, kaempferol-3-diglucoside, quercetin-7-glucoside [6], kaempferol 3-*O*-β-d-glucopyranosyl-(1→2)-*O*-α-l-xylopyranoside and kaempferol 3-*O*-α-l-rhamnopyranosyl-(1→2)-*O*-α-l-arabinopyranoside [7]. 

Hsouna and coworkers investigated the phytochemical composition of the essential oil from *L. maritima* aerial parts [8]. A number of oxygenated monoterpenes and monoterpene hydrocarbons have been identified. The authors also evaluated the essential oil in vivo antioxidant activity together with the in vitro anti-inflammatory effects on lipopolysaccharide (LPS)-stimulated RAW 264.7 cells. 

However, the potential health benefits of this plant species have not been properly investigated yet. Here, we aimed to investigate the phytochemical composition and the biological properties of the methanolic extract and sub-fractions of *L. maritima* aerial part. To the best of our knowledge, just the radical scavenging potency of the methanolic and aqueous extracts of *L. maritima* from two different sites of collection in Algeria has been previously described [9]. In our work, the in vitro antioxidant potential, the ability to inhibit nitric oxide production, and the pancreatic lipase inhibitory activity of *L. maritima* extract and sub-fractions were investigated. The metabolic and the immune systems play a pivotal role in survival, and they are strictly dependent on each other. Dysfunctions of such complex homeostatic mechanisms can lead to a cluster of chronic metabolic disorders, such as obesity, type 2 diabetes, and cardiovascular diseases, whose treatment constitutes nowadays the greatest challenge of research to protect global human health and welfare. Inflammatory processes are involved in obesity and type 2 diabetes: To indicate this metabolically triggered inflammation—caused by nutrients and metabolic surplus—which do not show the classic feature of inflammation, the new term “metainflammation” has been introduced [10].

According to these considerations, the pancreatic lipase inhibitory activity of *L. maritima* methanolic extract and its sub-fractions has also been taken into account, in order to highlight a potential use in body weight control. 

Moreover, the phytochemical profile has been elucidated with Gas Chromatography-Mass Spectrometry (GC-MS), High-Performance Liquid Chromathography with Dioide-Array Detector (HPLC-DAD), High-Performance Liquid Chromathography-High Resolution Mass Spectrometry (HPLC-HRMS), and Electrospray Ionization Tandem Mass Spectrometry (ESI-MS/MS) analyses.

## 2. Results

### 2.1. Phytochemical Profile

The apolar and polar constituents of a methanolic extract and its sub-fractions from the aerial parts of *L. maritima* were identified with GC-MS and HPLC-DAD, HPLC-HRMS, and ESI-MS/MS analyses. Table 1 reports the apolar compounds detected in the *n*-hexane (*n*-Hex) fraction. 

Nine fatty acids such as capric, 9-oxononanoic, lauric, 14-methylpentadecanoic, palmitic, myristic, stearic, 16-methyloctadecanoic acids, and 3-hydroxypropyl oleate were identified, being myristic and palmitic acids the most abundant ones (1.2% and 1.1% of total peak areas in TIC (Total Ion Current). Three terpenoids were also found, the monoterpene dihydroactinidiolide (0.2%), the diterpene neophytadiene (0.5%), and the triterpenoid β-amyrin acetate (1.8%). Among phytosterols, β-sitosterol was the major constituent (3.2%), followed by 24-methylenecycloartanol (2.7%) and tremulone (1.3%).

The analysis of the dichloromethane (DCM) sub-fraction allowed the identification of other interesting compounds (Table 2), among which the acyclic diterpene alcohol phytol and the monoterpene lactone loliolide were particularly abundant (8.4% and 5.3%, respectively). Two phenolic compounds, 2,4-di-tert-butylphenol (1.6%) and vanillic acid (0.5%), and the alkaloid methylethylmaleimide (0.6%) were also recognized in this fraction.

The total phenolic and total flavonoid content of *L. maritima* raw extract was also assessed. Their amounts were expressed in terms of chlorogenic acid and quercetin equivalents per g of dry material and were equal to 86.2 ± 0.8 mg/g and 17.85 ± 0.04 mg/g, respectively.

**Figure 1:**
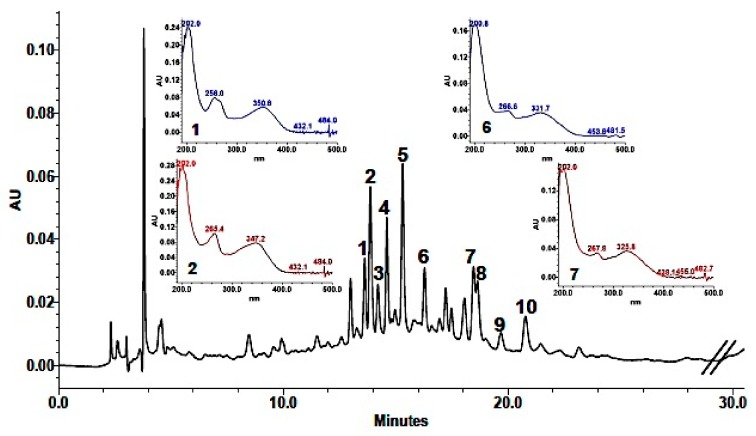
High-Performance Liquid Chromathography with Dioide-Array Detector, HPLC-DAD (310 nm) chromatogram of *L. maritima* methanolic extract.

Phenolic composition in the methanolic extract (MeOH) was achieved by a combination of analytical data from HPLC-DAD, UV, HPLC-HRMS, and coelution with authentic available compounds. As shown in Table 3, 10 major components were identified. Based on their UV spectra showing two major absorption peaks in the range of 240 to 280 nm (A-ring, benzoyl system, Band I) and 330–380 nm (B-ring, cinnamoyl system, Band II) the presence of flavonols or flavones in the extract was established. Analysis of the spectroscopic data suggested that HPLC eluted components were derivatives of the two aglycones quercetin (256, 301 *sh*, 370 nm) and kaempferol (265, 294 *sh*, 323 *sh*, 364 nm). This was also supported by the fragmentation pattern in the mass spectra (Table 3), in which quercetin derivatives were characterized by the [Aglycone + H]^+^ ion at *m*/*z* 303, while kaempferol derivatives showed an [Aglycone + H]^+^ ion at *m*/*z* 287. In addition, as reported for other Brassicaceae [11,12,13,14,15,16], they were mono-, bi- e tri-glycosides with sophorose (β-1,2-linked glucose) and rutinose (rhamnosyl-(α1→6)–glucose) as the most common disaccharide moieties (Table 3). Detected fragments of ions of sugars (−162 or −146 Da) also indicated that they were all O-glycosides. In addition, the hypsochromic shift of Band I compared with that of the aglycones revealed a substitution at 3- or 3,7-positions in the molecule, which allowed the identification of compounds with UV maxima around 356 and 350–354 nm as quercetin glycosides and compounds with UV maxima centered around 266 and 350–354 nm as kaempferol glycosides. Moreover, losses of 206 and 176 fragments suggested that some of these phenols were acylated with sinapic and ferulic acid, respectively. HPLC-DAD chromatogram is shown in Figure 1. 

Quantification indicated that the most abundant phenolic constituents of the methanolic extract from the aerial part of *L. maritima* were the four kaempferol derivatives: Kaempferol-3-*O*-β-sophoroside-7-*O*-α-rutinoside (0.24 ± 0.007 mg/mL extract), kaempferol-3-*O*-β-sophoroside-7-*O*-α-rhamnoside (0.19 ± 0.02 mg/mL extract), kaempferol-3-*O*-β-rutinoside-7-*O*-α-rhamnoside (0.28 ± 0.0002 mg/mL extract) and kaempferol-3-*O*-β-(sinapoyl)-sophoroside-7-*O*-α-rhamnosyl glucoside (0.10 ± 0.004 mg/mL extract). The other identified compounds were all present in smaller amounts (in the range of 0.01 to 0.07 mg/mL extract).

The aerial parts of *L. maritima* have also been investigated for their content of glucosinolates (GLSs), the typical sulfur metabolites produced by Brassicaceae [11,17]. HPLC analysis showed the presence of only one compound, which, based on its mass spectra fragmentation (316 *m*/*z*, [M + Na]^+^; 185 *m*/*z* (3) [(M + Na) – RCNOH − H_2_S]+; 154 *m*/*z* (2) [(M + Na) − Glu]+; 72 *m*/*z* (10) [CH_2_NCS]+; 58 *m*/*z* (19) [NCS]^+^), UV (225.4 nm) and HPLC elution with an authentic compound, which was identified as the aliphatic gluconapin (3-buteny-GLS). Quantitation against the calibration curve indicated that gluconapin amounted to 2.92 × 10^−5^ (±4.08 × 10^−7^) µmol/g of drug.

### 2.2. Antioxidant Activity

The radical scavenging activity of *L. maritima* methanolic raw extract and its sub-fractions was assessed with the 2,2-diphenyl-1-picrylhydrazyl (DPPH) colorimetric assay. An IC_50_ value equal to 937.70 ± 8.07 µg/mL was observed for the methanolic extract, while the ethyl acetate fraction was more effective, with an IC_50_ value of 253.81 ± 1.01 µg/mL (Table 4). These two samples demonstrated a better DPPH scavenging potency compared to the methanolic (IC_50_ range from 5.16 to 9.33 mg/mL), and aqueous (IC_50_ range from 14.87 to 74.17 mg/mL) extracts of *L. maritima* collected wild from two different sites of collection in Algeria tested by Asmaa and coworkers [9]. 

The DPPH test is a useful tool for the analysis of the free radical scavenging potency of antioxidants, but this model does not use a biologically relevant oxidizable substrate. Therefore, the antioxidant activity of our extracts was also verified with the β-carotene bleaching method, in which lipid substrates (Tween-emulsified linoleic acid with β-carotene) was used to determine the biological activity [18].

The ethyl acetate (EtOAc) fraction demonstrated a good biological activity, with an IC_50_ value equal to 25.15 ± 0.17 µg/mL after 30 min of incubation and to 31.37 ± 0.14 µg/mL after 60 min (Table 4). The DCM fraction also showed an IC_50_ value of 46.16 ± 0.91 µg/mL after 30 min, while a lower antioxidant potential was observed for the raw methanolic extract (IC_50_ = 72.91 ± 1.91 and 94.55 ± 4.70 µg/mL after 30 and 60 min, respectively).

### 2.3. Nitric Oxide Production Inhibition

**Figure 2:**
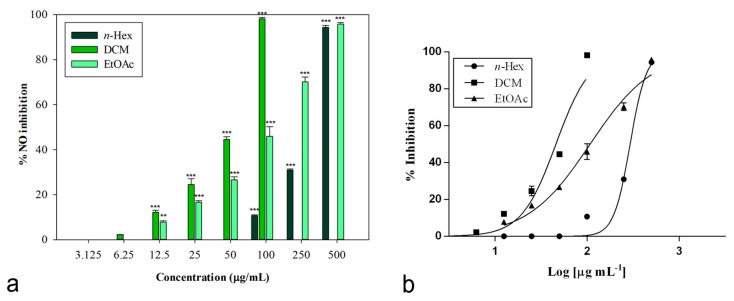
(**a**) Nitric oxide production inhibition induced by extracts from *L. maritima*. Data were expressed as means ± S.E.M. (*n* = 4). Mean values significantly different from the control were denoted with ** *p* < 0.01, *** *p* < 0.001 (Dunnett’s test). (**b**) Non-linear regression analyses.

The inhibitory potential of *L. maritima* methanolic extract and its sub-fractions on NO production was determined in vitro in LPS-stimulated RAW 264.7 macrophages. Nitrite, being a stable oxidized product of NO, was used to detect its production. The presence of nitrite was verified in cell culture medium by means of the Griess reagent. Cells were treated with different concentrations of *L. maritima* raw extract and fractions, ranging from 12.5 to 500 μg/mL, with the only exception of the DCM fraction. For this sub-extract, a range of lower concentrations (3.125 to 100 μg/mL) were used, as higher concentrations (500 and 250 μg/mL) caused cytotoxic effects on the macrophage cell line. At a concentration of 100 μg/mL, the DCM fraction was able to induce 98.14% ± 0.60% inhibition of NO production (*p* < 0.001 compared to control, Dunnett’s multiple comparison test), without showing any cytotoxic effect (Figure 2). The other two sub-extracts (*n*-hexane and ethylacetate) were able to induce an inhibition percentage of about 90% at the highest concentration tested (500 μg/mL). 

The raw MeOH extract was not effective, while its sub-fractions significantly reduced LPS-induced synthesis of NO in a concentration-dependent manner. Table 5 reports obtained IC_50_ values. The *n*-hexane fraction showed the lowest inhibitory activity (IC_50_ = 298.80 ± 2.52 μg/mL). An IC_50_ value equal to 107.80 ± 7.99 μg/mL was observed for the ethyl acetate fraction.

An excellent biological activity was observed for the DCM extract, with an IC_50_ value equal to 45.86 ± 1.05 μg/mL (Figure 2). This result is significant if compared to both indomethacin (IC_50_ = 58.00 ± 0.90 μg/mL) and the known nitric oxide synthase inhibitor L-NAME (IC_50_ = 45.86 ± 0.46 μg/mL), used as positive controls (*p* < 0.05, Bonferroni post-hoc test). 

### 2.4. Anti-Obesity Activity

**Figure 3:**
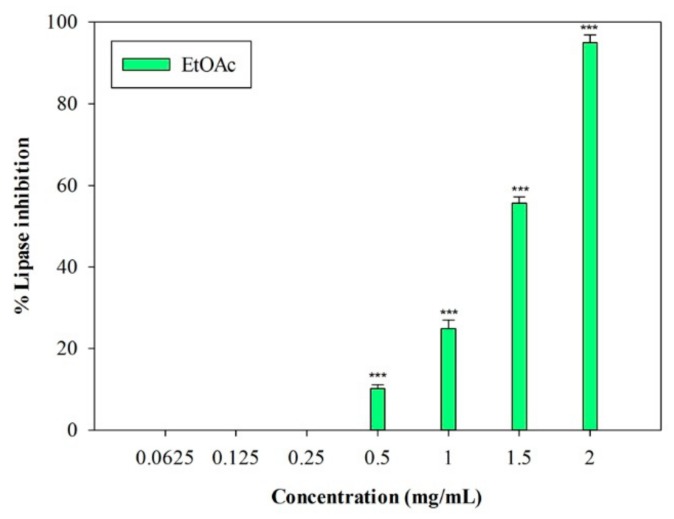
Concentration-dependent pancreatic lipase inhibition induced by EtOAc sub-fraction of *L. maritima* methanolic extract. Data were expressed as means ± S.E.M. (n = 4). *** *p* < 0.001 compared to control (Dunnett’s test).

An in vitro porcine pancreatic lipase inhibitory test was carried out. In this preliminary study, the inhibition of lipase activity was evaluated through a colorimetric method, by monitoring the hydrolysis of 4-nitrophenyl caprylate (*p*-NPC), which releases the yellow chromogen *p*-nitrophenol. The well-known pancreatic lipase inhibitor Orlistat [19] was used as a positive control (IC_50_ = 0.018 ± 0.001 mg/mL). The raw MeOH extract and the *n*-Hex and DCM fractions were not active at the highest concentration tested (2 mg/mL). On the contrary, the EtOAc fraction showed inhibitory activity, with an IC_50_ value equal to 1.33 ± 0.03 mg/mL (Figure 3). At the highest concentration (2 mg/mL), this fraction induced 95.02% ± 1.83% inhibition of the enzyme, and an inhibition percentage equal to 55.70% ± 1.46% was detected at 1.5 mg/mL (*p* < 0.001, Dunnett’s multiple comparison test).

## 3. Discussion

Once the plant material was extracted with MeOH at room temperature, the obtained raw extract was fractionated using solvents with increasing polarity in order to achieve a first separation of the chemical components.

Our phytochemical analyses were carried out by using GC-MS, HPLC-DAD, HPLC-HRMS, and ESI-MS/MS analyses. The less apolar compounds include a number of fatty acids, such as myristic and palmitic acids, terpenoids, such as dihydroactinidiolide, neophytadiene, and β-amyrin acetate, and some phytosterols, such as β-sitosterol, 24-methylenecycloartanol, and tremulone. The DCM fraction was characterized by the presence of phytol, loliolide, methylethylmaleimide, and two phenolic compounds, 2,4-di-tert-butylphenol and vanillic acid. A combination of analytical data from HPLC-DAD, UV, and HPLC-HRMS allowed the identification of the most polar components. Four kaempferol derivatives were the most abundant phenolics: Kaempferol-3-*O*-β-sophoroside-7-*O*-α-rutinoside, kaempferol-3-*O*-β-sophoroside-7-*O*-α-rhamnoside, kaempferol-3-*O*-β-rutinoside-7-*O*-α-rhamnoside, and kaempferol-3-*O*-β-(sinapoyl)-sophoroside-7-*O*-α-rhamnosyl glucoside.

Moreover, as Brassicaceae are typically characterized by the presence of glucosinolates [20], the identification of these sulfur metabolites was also carried out. Analyses allowed the detection of the presence of gluconapin.

Our findings are in accordance with those of Fiorentino and co-workers [7], who identified kaempferol glycosides from *L. maritima* collected in a coastal area in Naples (Southern Italy). Some anthocyanins have also been previously identified in this species [21].

The aim of our work was also to study the biological properties of *L. maritima* MeOH extract and its sub-fractions: The in vitro antioxidant potential, the ability to inhibit nitric oxide production, and the pancreatic lipase inhibitory potential were investigated. 

The radical scavenging potency was tested by means of the DPPH test. The EtOAc fraction and the raw extract were effective, with IC_50_ values equal to 253.81 ± 1.01 µg/mL and 937.70 ± 8.07 µg/mL, respectively. Our results differ from those reported by Asmaa and coworkers [9], who investigated the radical scavenging potency of *L. maritima* MeOH extracts from Algeria with the same test, reporting IC50 values much higher, ranging from 5.16 to 9.33 mg/mL.

The EtOAc fraction showed a good biological activity also in the second test, the β-carotene bleaching method, with IC50 values equal to 25.15 ± 0.17 µg/mL and to 31.37 ± 0.14 µg/mL after 30 and 60 min of incubation, respectively. The test was performed after 30 and 60 min to evaluate how the antioxidant potential varied with increasing exposure time to heat-induced degradation. The activity of MeOH extract and DCM fraction decreased over time (Bonferroni post-hoc test), while no statistical differences were detected between 30 and 60 min for the EtOAc fraction, whose effectiveness remains unchanged over time.

Moreover, the ability to inhibit nitric oxide production was evaluated in the RAW 264.7 macrophage cell line stimulated with LPS. Nitric oxide is a signaling molecule that plays a pivotal role in the regulation of cardiovascular, nervous, and immunological systems. However, this molecule is also a free oxygen radical, which can exert cytotoxic effects in pathological processes such as inflammatory disorders. Therefore, the inhibition of its overproduction by iNOS can be useful in the treatment of inflammatory conditions [22].

Nitric oxide production was quantified by the chromogenic Griess reaction, measuring the presence of nitrite, a stable nitric oxide metabolite [23]. All the *L. maritima* sub-extracts significantly affected LPS-induced synthesis of NO in a concentration-dependent manner, being the DCM fraction the most effective sample. An IC_50_ of 45.86 ± 1.05 μg/mL was obtained, a significant value compared to the positive controls indomethacin and L-NAME.

The inhibitory activity on pancreatic lipase, a key enzyme for the absorption of dietary fats, has also been taken into account in our study, in order to have some preliminary information about the potential anti-obesity properties of this plant species. 

Some recent findings evidenced a link between antioxidant status and obesity. An excess of body weight may cause enhanced oxidative stress and generated free radicals may play a role in the development of obesity-related co-morbidities. In this context, antioxidant compounds may exert protective effects against these pathological conditions [24]. A number of studies have demonstrated the potential health benefits in the treatment of obesity of natural antioxidant compounds such as flavonoids, as well as their action on various molecular targets [25].

In our study, the inhibition of pancreatic lipase was assessed in vitro through a colorimetric method based on the use of 4-nitrophenyl caprilate as a substrate. The EtOAc polar fraction of *L. maritima* proved to be effective in inhibiting the enzyme, with an IC_50_ value equal to 1.33 ± 0.03 mg/mL.

## 4. Materials and Methods

### 4.1. Chemicals and Reagents

2,2-Diphenyl-1-picrylhydrazyl (DPPH), 4-nitrophenyl caprylate (*p*-NPC), aluminum chloride, anion exchange resin Sephadex DEAE A-25, ascorbic acid, Dulbecco’s modified Eagle’s medium (DMEM), fetal bovine serum (FBS), Folin-Ciocalteu reagent, Griess reagent, indomethacin, L-glutamine, penicillin/streptomycin, linoleic acid, lipase type II from porcine pancreas, MTT tetrazolium salt, N^G^-nitro-L-arginine methyl ester (L-NAME), Orlistat, partially purified Helix pomatia Type-1 sulfatase, phosphate-buffered saline (PBS), phosphomolybdic acid hydrate, propyl gallate, sodium acetate, trypan blue, Tween 20, and β-carotene were obtained from Sigma-Aldrich S.p.A. (Milan, Italy). Isoquercitrin (quercetin-3-*O*-β-glucoside) and kaempferol-3-*O*-β-rutinoside were obtained by Phytolab, Italy. Aluminum TLC silica gel 60 F254 plates were purchased from Merck KGaA. Gluconapin (3-butenylglucosinolate) was supplied by C2 Bioengineering Aps in approximately 90–100% pure grade. Poly-Prep^®^ chromatography columns were obtained from Bio-Rad Laboratories. Murine macrophage cell line RAW 264.7 was obtained from Type Culture Collection (ATCC) no. TIB-71, UK. Methanol and water (HPLC grade) were purchased from J.T. Baker and the other solvents used (reagent grade) were purchased from VWR International s.r.l. (Milan, Italy). 

### 4.2. Plant Material and Extraction

Wild *L. maritima* (L.) Desv. aerial parts were collected in September in Calabria, in the Reggio Calabria district (leg. F. Conforti, det. F. Conforti). A voucher specimen was deposited in the Herbarium of the University of Calabria (CLU 22691). Dried plant material (65.5 g) was extracted through maceration with MeOH at room temperature (plant material to solvent ratio 1:10 g/mL, 48 h × 3 times). Obtained solutions were filtered and dried under reduced pressure (extraction yield 15.2%). A fraction of this raw extract was then suspended in MeOH/water (9:1) and extracted with *n*-Hex (yield, 1.4%. referred to dry plant material). The residue aqueous methanol was then evaporated under reduced pressure, suspended in distilled water, and extracted twice successively, first with DCM and then with EtOAc (1.4% and 0.3% yield, respectively). Samples were kept at −20 °C.

### 4.3. Total Phenolic Content and Flavonoid Content

The total phenolic content was determined using the Folin Ciocalteau reagent as described by Singleton and Rossi [26] with a few modifications, as previously reported [27]. The raw extract (2 mg/mL in acetone/methanol/water/formic acid, 40:40:20:0.1 *v*/*v*/*v*/*v*), 200 μL, was mixed with 1 mL of Folin-Ciocalteau reagent and 1 mL of sodium carbonate solution (7.5% *w*/*v*). The mixtures were allowed to stand at room temperature for 2 h. The absorbance was measured at 726 nm. 

Total flavonoid content was estimated using an aluminum chloride colorimetric method previously described [16]. *L. maritima* extract (2 mg/mL in 80% ethanol), 1 mL, was mixed with 1 mL of 2% aluminum chloride solution. The mixture was allowed to stand at room temperature for 15 min. The absorbance of the mixture was then measured at 430 nm.

Results were calculated from calibration curves based on the standards such as chlorogenic acid (analysis of phenolics) or quercetin (flavonoids determination) and were expressed as mg of chlorogenic acid or quercetin equivalent per g of dry plant material, respectively.

### 4.4. GC-MS Analysis

Gas chromatography-mass spectrometry (GC-MS) analyses of the *n*-Hex and DCM fractions were carried out using a Hewlett-Packard 6890 gas chromatograph. The equipment had a SE-30 capillary column (dimethylpolysiloxane 100%, 0.25 mm in diameter, 30 m length, 0.25 µm film thickness) and was coupled to a mass spectrometer Hewlett Packard 5973. A programmed temperature from 60 to 280 °C was used to perform the analyses, as previously described [28]. Metabolites were identified by using the Wiley 138 mass spectral library of the GC-MS system.

### 4.5. Analysis of Phenolics

#### 4.5.1. TLC

Content of phenolics in the MeOH extract from *L. maritima* was determined following the general procedure reported in Marrelli et al. [16]. A preliminary screening was carried out by TLC eluted with EtOAc:HCOOH:CH_3_CO_2_H:H_2_O (100:11:11:27, *v*:*v*). Components were visualized with phosphomolybdic acid reagent (10% EtOH) or alternatively with Natural Products-Polyethyleneglycol reagent (NP-PEG, Sigma). TLC plates were dried off at 110 °C and then observed under visible-254 nm or in UV-366 nm light.

#### 4.5.2. HPLC-DAD

HPLC analyses were performed with the same apparatus and analytical column, as previously described [16]. Analytical conditions were as follows: Solvent A, H_2_O-HCOOH 0.1%, pH 2.7; solvent B, CH_3_CN-HCOOH 0.1%; elution gradient, 10–60% B in 60 min; flow rate, 1 mL/min. UV spectra of eluted components were conventionally recorded at 210, 270, 310, and 350 nm. All analyses were run in triplicate. HPLC identification was based on coelution of available phenolics run in the same analytical conditions and comparison of their RT and UV spectra; chromatographic and spectroscopic data from the literature were also used [7,15,16,29,30,31].

The external standard method was employed for quantitation. A 7-level calibration (0.0078–0.5 mg/mL MeOH) curve was created by injection of isoquercitrin (quercetin-3-*O*-ß-glucoside) and kaempferol-3-*O*-ß-rutinoside according to [16]. The correlation coefficient (r^2^) of the standard curve in the linear plot was r^2^ = 0.999 (y = 5 × 10^0.7x^ + 166,437) for isoquercitrin and r^2^ = 0.999 (y = 3 × 10^0.7x^ + 27,270) for kaempferol-3-*O*-ß-rutinoside, indicating a good linearity between peak areas and concentrations within the tested concentration range.

The precision of the adopted HPLC method was determined by calculation of the intra-day % RSDs of retention times (0.81% to 1.04% for isoquercitrin; 0.18% to 1.29% for kaempferol-3-*O*-ß-rutinoside) and peak areas (3.83% to 1.86% for isoquercitrin; 2.85% to 1.12% for kaempferol-3-*O*-ß-rutinoside). Calculated % RSDs for inter-day retention times were 0.39% (isoquercitrin) and 0.23% (kaempferol-3-*O*-ß-rutinoside), while for peak areas they were 4.93% (isoquercitrin) and 1.29% (kaempferol-3-*O*-ß-rutinoside). The limit of detection, LoD, was 0.03 µg/mL and 0.19 µg/mL for isoquercitrin and kaempferol-3-*O*-ß-rutinoside, respectively; limit of quantification, LoQ, was 0.12 µg/mL and 0.59 µg/mL for isoquercitrin and kaempferol-3-*O*-ß-rutinoside, respectively. All HPLC analyses for identification and quantitation of phenolics were run in triplicate.

#### 4.5.3. HPLC-HRMS

A benchtop single-stage mass spectrometer (Exactive) equipped with a heated electrospray ion source HESI II and coupled to an Accela HPLC system (Thermo Fisher Scientific, Waltham, MA, USA) was used. Analytical conditions were the same as described in reference [16]. Xcalibur software (v2.1.0; Thermo Fisher) was used for data acquisition and processing. Structural characterization was based on comparison with mass spectrometric data of available reference phenolics and/or mass spectra from library files [7,15,16,29,30,31].

### 4.6. Analysis of Glucosinolates

#### 4.6.1. Extraction and Desulfation

Finely pulverized dried aerial parts of *L. maritima* (200 mg) were extracted (ISO-Method 9167-1, 1992) with boiling MeOH:H_2_O (70:30%) to isolate glucosinolates (GLSs). Extraction and desulfation with sulfatase (*H. pomatia* type-1) were carried out in duplicate following the procedure already described [16,32]. TLC (iPrOH:EtOAc:H_2_O, 7:1:2, *v*/*v*) was used to monitor GLSs elution and desulfation; products were first visualized under UV light at 254 and 366 nm and then revealed by spraying TLC plates with phosphomolybdic acid reagent (10% EtOH).

#### 4.6.2. Chemical Analysis and Quantitation

Desulfoglucosinolates (DGLSs) were analyzed by HPLC, according to Argentieri et al. [33] on a Waters 600 HPLC system equipped with a Photodiode-Array-Detector, PDA 2998. DGLSs separation was afforded with a Phenomenex Gemini-C18 reversed phase column (250 × 4.6 mm, 5 µm particle size), equipped with a Security Guard-C18 cartridge (4 mm × 3 cm, 5 µm particles), using the following elution system: Solvent A, H_2_O; solvent B, MeOH. (1.5% B in A, up to 60% of B at 50 min and 100% of B at 54 min; flow rate, 1 mL/min). Data were processed with EmpowerTM 2 Waters Software. UV spectra at 229 nm were recorded for each component eluted. RT and UV spectra of available authentic DGLSs, run in the same analytical conditions, were also compared for identification. 

Quantitation was achieved by the external standard method. A set of known concentrations (0.001–0.5 mg/mL) of desulphogluconapin in MeOH was used to create a calibration curve. Triplicate measurements were taken for each level in the standard curve. Linearity was determined by plotting the peak area ratio (y) recorded for each GLSs vs. its concentration (x). The calibration curve was fitted to a linear function with the calibration coefficient r^2^ = 0.999 (y = 1 × 10^0.7x^ + 13,454) for desulphogluconapin indicating a good linearity between peak areas and concentrations within the tested concentration range. Precision of the HPLC method was determined by repeatability (intra-day RSD % values of retention times and peak areas from 3 injections of the reference analyte desulphogluconapin in the same day) and intermediate precision (inter-day precision obtained by comparing the results on 3 consecutive validation days). Calculated RSDs intra-day values were from 0.82% to 0.22% for retention times and from 1.24% to 1.04% for peak areas. Calculated RSDs for inter-day retention times was 0.90%, while for peak areas, it was 0.27%. The limit of detection (LoD) and limit of quantification (LoQ) were evaluated on the basis of the signal-to-noise ratios (S:N) of 3:1 and on 10:1, respectively. The LoD was 0.16 µg/mL, while the LoQ was 0.25 µg/mL for desulphogluconapin. Three replicates of all the HPLC analyses for identification and quantitation of GLSs were run.

#### 4.6.3. ESI-MS/MS Analysis

An 1100 Series Agilent LC/MSD Trap System VL equipped with an Agilent Chem-station (LC/MSD Trap-Software 4.1) was used to run the analyses and for processing of data. Settings of the ESI ion source type, both in negative and positive mode, were as follows: Capillary voltage, 400 V; nebulizer gas (N2), 15 psi; drying gas (N2), heated at 350 °C and introduced at a flow rate of 5 L/min. Full scan spectra were acquired over the range of 100 to 2200 with a scan time of 13.000 m/z/s. Mass/MS fragmentation was performed as previously described [16]. Identification was based on comparison with available reference mass spectra from standard GLSs and/or by coelution with authentic samples.

### 4.7. Biological Assays

#### 4.7.1. DPPH Radical Scavenging Activity

The radical scavenging activity was assessed spectrophotometrically, as previously described [34]. Briefly, 0.8 mL of a 1 × 10^−4^ methanolic solution of 2,2-diphenyl-1-picrylhidrazyl (DPPH) was added to 0.2 mL of graded concentration of samples (ranging from 5 to 1000 µg/mL). After 30 min of incubation in the dark at room temperature, absorbance was measured at 517 nm. Ascorbic acid was used as a positive control, and experiments were performed in triplicate.

#### 4.7.2. Linoleic Acid/β-Carotene Bleaching Assay

The antioxidant activity of the raw extract and its fractions was evaluated using the β-carotene bleaching assay [35]. Briefly, a β-carotene solution (1 mL, 0.5 mg/mL in CHCl_3_) was added to linoleic acid (0.02 mL) and 100% Tween 20 (0.2 mL). Chloroform was then removed, followed by the addition of distilled water (100 mL). An aliquot of 5 mL of the obtained emulsion was transferred into tubes containing 0.2 mL of different samples solutions (0.25–100 μg/mL), and the mixtures were placed in a water bath at 45 °C; absorbance was measured at 470 nm at the initial time and after 30 and 60 min. The successful prevention of β-carotene bleaching was used to measure the antioxidant activity. The experiments were carried out in triplicate. Propyl gallate was used as a positive control.

#### 4.7.3. Inhibition of Nitric Oxide Production in LPS-Stimulated RAW 264.7 Cells

Murine macrophage RAW 264.7 cells stimulated with LPS were used to assess the ability of extracts to inhibit NO production. Cells were maintained in Dulbecco’s Modified Eagle’s Medium (DMEM) supplemented with fetal bovine serum (FBS, 10%), L-glutamine (1%), and antibiotic solution (penicillin/streptomycin, 1%) under 5% CO_2_ at 37 °C. Cells were removed from the culture flask by scraping. Cell counts were performed using a standard trypan blue cell counting technique. Cells were then seeded onto 96 well-plates (1 × 10^5^ cells/well in 100 µL DMEM per well) and cultured for 24 h. The medium was then removed and replaced with fresh DMEM containing 1 μg/mL LPS and samples (3–500 μg/mL in dimethyl sulfoxide, DMSO to medium final ratio 0.5% *v*/*v*). After 24 h of incubation, the presence of nitrite, a stable product of NO oxidation, was determined in cell culture media with the Griess reagent [27]. Briefly, cell culture supernatant was collected and mixed with an equal volume (100 μL) of Griess reagent. Absorbance was measured at 550 nm. Indomethacin and the nitric oxide synthase inhibitor N^G^-nitro-l-arginine methyl ester (L-NAME) were used as positive controls.

The 3-(4,5-dimethylthiazol-2-yl)-2,5-diphenyltetrazolium bromide (MTT) assay [36] was used to verify the absence of cytotoxic effects on treated cells. Briefly, RAW 264.7 cells were incubated with 0.5% *w*/*v* MTT (100 μL/well). Four hours later, dimethyl sulfoxide (100 μL/well) was added to dissolve the formazan crystals, and absorbance was measured at 550 nm.

#### 4.7.4. Pancreatic Lipase Assay

The pancreatic lipase inhibitory effects of *L. maritima* was assessed using the method previously described [16], with some modifications, using 4-nitrophenyl caprilate (*p*-NPC, 5 mM in DMSO) as a substrate. The enzyme solution (1 mg/mL) was prepared by suspending porcine pancreatic lipase in distilled water. This solution (25 µL) was added to NPC (25 µL), Tris-HCl buffer (1 mL), and *L. maritima* samples (25 µL, concentrations ranging from 2 to 0.06 mg/mL). The mixtures were then incubated at 37 °C for 25 min, and the absorbance was measured at 412 nm. Orlistat (20 μg/mL) was used as a positive control.

### 4.8. Statistical Analysis

Experiments on cell cultures were run in quadruplicate. For other assays, 3 replicates were performed. Data were expressed as means ± S.E.M. D’Agostino-Pearson’s K2 test and Levene’s test were used for assessing normality of data and homogeneity of variances, respectively. Raw data were fitted through nonlinear regression to calculate the IC_50_ values using the Graph-Pad Prism Software (San Jose, CA, USA). One way analysis of variance (ANOVA) with the Bonferroni and the Dunnett’s multiple comparison post-hoc tests were performed using SigmaStat Software (Jantel Scientific Software, San Rafael, CA, USA).

## 5. Conclusions

A close relationship between obesity and the imbalance of immunity and inflammation mediators has been discovered. The inflammatory response caused by obesity is similar to the classical inflammatory response, as it is characterized by an increase of inflammatory cytokines and of leukocytes recruitment. On the other hand, the nature of obesity-induced metainflammation is unique because it involves several organs. Inflammation plays a pivotal role in a number of obesity-related morbidities [37,38].

Plants can potentially represent a source of a large number of bioactive compounds useful for weight control [39,40,41]. Many phytochemicals, acting through different mechanisms, have been proven to be effective in body weight management [42,43]. The inhibition of pancreatic lipase enzyme is one of the major mechanisms taken into account in the search for new drugs [44,45]. 

At the same time, phytochemicals are also attractive due to their anti-inflammatory potential and the chance to find new drugs that could be an alternative to conventional steroidal and non-steroidal anti-inflammatory drugs (NSAIDs), whose side effects are well known [46,47].

*L. maritima* is primarily grown as an ornamental plant. However, it has a history of use as a diuretic, antiscorbutic, and as an astringent in the treatment of gonorrhea. Moreover, in Southern Italy, this species is used as a folk remedy against abdominal pains and coughs [48].

Our findings highlight interesting in vitro biological activities for the understudied species *L. maritima*. A number of polar and apolar compounds from the aerial parts of this plant species collected wild in Southern Italy have been identified. The EtOAc sub-fraction of the MeOH extracts showed a good in vitro inhibitory activity on pancreatic lipase. To the best of our knowledge, this is the first report on the anti-obesity potential of this perennial herb. Moreover, a good antioxidant potential was demonstrated for the methanolic raw extract and its sub-fractions, and all the sub-extracts were effective in inhibiting the production of nitric oxide in a murine macrophages cell line used as a model. The dichloromethane fraction was particularly interesting if compared to the positive controls. These data provide the basis for further research that could corroborate these first results on the potential health benefits of *L. maritima* extracts and its constituents and lead to the identification and characterization of the single active principles most responsible for the observed biological activity.

## Figures and Tables

**Table 1:** Phytochemical profile of the *n*-hexane (*n*-Hex) sub-fraction of *L. maritima* (L.) Desv.

Compound	Rt ^1^	RAP ^2^
**Fatty acids**		
Capric acid	13.084	Tr ^3^
9-Oxononanoic acid	13.833	0.1
Lauric acid	15.039	0.1
14-Methylpentadecanoic acid	18.119	0.2
Palmitic acid	18.136	1.1
Myristic acid	18.428	1.2
Stearic acid	19.622	0.2
16-Methyloctadecanoic acid	19.634	0.1
3-Hydroxypropyl oleate	21.194	0.4
**Terpenes**		
Dihydroactinidiolide	14.976	0.2
Neophytadiene	17.468	0.5
β-Amyrin acetate	37.397	1.8
**Phytosterols**		
β-Sitosterol	34.065	3.2
Tremulone	36.071	1.3
24-Methylenecycloartanol	38.089	2.7
**Others**		
2,6,10,14-Tetramethylheptadecane	14.090	tr
Cyclododecane	16.102	tr
Phytone	17.525	1.0
Eicosane	22.040	0.6
Heptacosane	23.474	0.9
Hexacosane	24.983	0.7
Cyclotetracosane	25.749	0.9

^1^ Retention time (min). ^2^ Relative peak area percentage. ^3^ Tr: Traces percentages < 0.1%.

**Table 2:** Phytochemical profile of the dichloromethane (DCM) sub-fraction of *L. maritima* (L.) Desv.

Compound	Rt ^1^	RAP ^2^
Benzoic acid	10.815	1.5
Methylethylmaleimide	11.604	0.6
2,4-Di-tert-butylphenol	14.644	1.6
Vanillic acid	15.193	0.5
Loliolide	17.148	5.3
Phytol	17.450	8.4

^1^ Retention time (as min). ^2^ Relative peak area percentage.

**Table 3:** Analytical data of flavonol glycosides identified in the methanolic extract of *L. maritima* aerial parts.

Peak *	Compound	UV (λ max, nm)	[M + H]^+^, *m*/*z*	MS/MS, *m*/*z*, (I%)
**1**	Quercetin-3-*O*-β-sophoroside-7-*O*-α-rutinoside	256.0, 266.3 *sh*, 350.8	935	773 (17) [M − 162 + H]^+^; 611 (62) [M − 162 − 162 + H]^+^; 449 (17) [M − 162 − 162 − 162 + H]^+^; 303 (14) [Aglycone + H]^+^
**2**	Kaempferol-3-*O*-β-sophoroside-7-*O*-α-rutinoside	265.4, 323.0 *sh*, 347.2	919	757 (24) [M − 162 + H]^+^; 611 (1) [M − 162 − 146 + H]^+^; 595 (93) [M − 162 − 162 + H]^+^; 433 (24) [M − 162 − 162 − 162 + H]^+^; 287 (22) [Aglycone + H]^+^
**3**	Quercetin-3-*O*-β-sophoroside-7-*O*-α-rhamnoside	256.0, 265.5 *sh*, 348.4	773	611 (17) [M − 162 + H]^+^; 449 (93) [M − 162 − 162 + H]^+^; 303(30) [Aglycone + H]^+^
**4**	Kaempferol-3-*O*-β-sophoroside-7-*O*-α-rhamnoside	265.4, 321.8 *sh*, 344.8	757	595 (17) [M − 162 + H]^+^; 433 (100) [M − 162 − 162 + H]^+^; 287 (38) [Aglycone + H]^+^
**5**	Kaempferol-3-*O*-β-rutinoside-7-*O*-α-rhamnoside	265.4, 321.8 *sh*, 344.8	741	595 (60) [M − 146]^+^; 433 (84) [M − 146 − 162]^+^; 287 (31) [Aglycone + H]^+^
**6**	Quercetin-3-*O*-β-(sinapoyl)-sophoroside-7-*O*-α-rhamnosyl-Glucoside	256.6, 266.6 *sh*, 331.7	1141	979 (18) [M − 162 + H]^+^; 611 (38) [M − 368 − 162 + H]^+^; 449 (13) [M − 162 − 206 − 162 − 162 + H]^+^; 303 (8) [Aglycone + H]^+^
**7**	Kaempferol-3-*O*-β-(sinapoyl)-sophoroside-7-*O*-α-rhamnosyl-glucoside	267.8, 320.8 *sh*, 325.8	1125	1095 (28) [M + H − 30]^+^; 963 (15) [M − 162 + H]^+^; 611 (1) [M − 162 − 162 − 192 + H]^+^; 595 (46) [M − 162 − 162 − 205 + H]^+^; 287(12) [Aglycone + H]^+^
**8**	Quercetin -3-*O*-β-(feruloyl)-sophoroside-7-*O*-α-rhamnoside	256.0, 270.1 *sh*, 331.7	949	449 (46) [M − 162 − 176 − 162 + H]^+^; 303 (12) [Aglycone + H]^+^
**9**	Kaempferol-3-*O*-β-(sinapoyl)-sophoroside-7-*O*-α-rhamnoside	266.6, 322.2	963	817 (1) [M − 146]^+^; 595 (6) [M − 162 − 206]^+^; 433 (47) [M − 162 − 206 − 162]^+^; 287 (14) [Aglycone + H]^+^
**10**	Kaempferol-3-*O*-β-(feruloyl)-sophoroside-7-*O*-α-rhamnoside	267.8, 324.6	933	771 (1) [M − 162 + H]^+^; 595 (5) [M − 162 − 176 + H]^+^; 433 (67) [M − 162 − 176 − 162 + H]^+^; 287 (21) [Aglycone + H]^+^

* For peak numbering refer to Figure 1.

**Table 4:** Antioxidant activity of *L. maritima* (L.) Desv. extract and fractions.

Sample	IC_50_ (µg/mL)		
	DPPH Test	β-Carotene Bleaching Test	
		30 min	60 min
MeOH extract	937.70 ± 8.07 ^c^	72.91 ± 1.91 ^d^	94.55 ± 4.70 ^e^
*n*-Hex	˃1000	˃100	˃100
DCM	˃1000	46.16 ± 0.91 ^c^	64.84 ± 1.18 ^d^
EtOAc	253.81 ± 1.01 ^b^	25.15 ± 0.17 ^b^	31.37 ± 0.14 ^b^
Ascorbic acid ^1^	2.00 ± 0.01 ^a^	-	-
Propyl gallate ^1^	-	1.00 ± 0.02 ^a^	1.00 ± 0.02 ^a^

Data are expressed as mean ± SEM (*n* = 3). Different letters along column (DPPH test) or between columns (β-carotene bleaching test) indicate statistically significant differences (Bonferroni post-hoc test, *p* < 0.05). ^1^ Positive controls.

**Table 5:** *L. maritima* (L.) Desv. inhibitory activity on NO production.

Sample	IC_50_ (µg/mL)
MeOH extract	n.a.
*n*-Hex	298.80 ± 2.52 ^c^
DCM	45.86 ± 1.05 ^a^
EtOAc	107.80 ± 7.99 ^b^
Indomethacin ^1^	58.00 ± 0.90 ^a^
L-NAME ^1^	45.86 ± 0.46 ^a^

Data are expressed as mean ± SEM (*n* = 3). Different letters indicate statistically significant differences (Bonferroni post-hoc test, *p* < 0.05). n.a. = not active at the highest concentration tested. ^1^ Positive controls.
